# Enhanced serum levels of tumor necrosis factor-α, interleukin-1β, and -6 in sarcopenia: alleviation through exercise and nutrition intervention

**DOI:** 10.18632/aging.205254

**Published:** 2023-11-29

**Authors:** Ke-Vin Chang, Wei-Ting Wu, Yu-Hsin Chen, Lan-Rong Chen, Wei-Hsiang Hsu, Yun-Lian Lin, Der-Sheng Han

**Affiliations:** 1 Department of Physical Medicine and Rehabilitation, National Taiwan University Hospital and National Taiwan University College of Medicine, Taipei, Taiwan; 2 Department of Physical Medicine and Rehabilitation, National Taiwan University Hospital, Bei-Hu Branch, Taipei, Taiwan; 3 Center for Regional Anesthesia and Pain Medicine, Taipei Municipal Wang-Fang Hospital, Taipei, Taiwan; 4 Department of Chinese Pharmaceutical Sciences and Chinese Medicine Resources, China Medical University, Taichung, Taiwan; 5 Department of Pharmacy, National Taiwan University, Taipei, Taiwan

**Keywords:** sarcopenia, inflammation, cytokine, exercise, nutrition

## Abstract

Background: Limited research has been conducted on the post-intervention inflammatory status in sarcopenic patients, despite previous studies revealing elevated pro-inflammatory markers. This study aimed to investigate the potential elevation of specific pro-inflammatory cytokines in sarcopenic patients and evaluate the effects of exercise and nutritional support interventions on these cytokine levels.

Methods: In this post-hoc analysis of a randomized controlled trial (RCT), 57 individuals with sarcopenia from the RCT and 57 non-sarcopenic participants from the same geriatric community cohort that did not participate in the RCT were enrolled. Grip strength and body composition measurements were recorded. Tumor necrotizing factor (TNF)-α, interleukin (IL)-1β, IL-6, and IL-15 levels were assessed at baseline for both groups and after a 12-week intervention consisting of resistive exercise and supplementation with branched-chain amino acids, calcium, and vitamin D3 in the patients with sarcopenia.

Results: The sarcopenic group demonstrated significantly lower body weight, body mass index, grip strength, and skeletal muscle mass index. Moreover, sarcopenic patients exhibited higher levels of TNF-α (*p*=0.007), IL-1β (*p*<0.001), and IL-6 (*p*<0.001), while no significant difference was observed in IL-15 (*p*=0.345) between participants with and those without sarcopenia. Following the intervention, the sarcopenic group experienced significant improvements in grip strength and skeletal muscle mass index with a notable reduction in TNF-α (*p*=0.003), IL-1β (*p*=0.012) and IL-6 (*p*=0.001) levels.

Conclusions: Sarcopenic patients exhibit elevated levels of TNF-α, IL-1β, and IL-6, which declined after nutrition support and exercise interventions. However, further research is necessary to evaluate the long-term impact of these interventions on cytokine levels.

## INTRODUCTION

Sarcopenia, which refers to loss of muscle mass, strength, and function pertinent to aging, affects a significant portion of the population aged 60 years and older, with a global prevalence ranging from 10% to 27% [1]. This condition has been associated with several negative health outcomes, including cognitive impairment [2], depression [3], an increased risk of falls [4], prolonged hospitalization [4], and poor prognosis for cancer patients [5]. Independent risk factors for sarcopenia include age, prefrailty, malnutrition, diabetes, and low physical activity [6].

The development of sarcopenia is believed to involve an imbalance between muscle protein synthesis and breakdown, as skeletal muscles continuously undergo deterioration. Skeletal muscles have been proposed as a central mediator between sarcopenia and immune senescence in the aging process [7]. Aging is characterized by a chronic state of slightly elevated plasma levels of pro-inflammatory mediators [8]. As people age, there is an increased number of cells exiting the cell cycle and entering cellular senescence. Senescent cells would be presented as a senescence-related secretory phenotype, which triggers the generation of pro-inflammatory mediators [7].

Studies have found elevated levels of markers such as C-reactive protein and erythrocyte sedimentation rate in sarcopenic patients, particularly in an aging hip fracture cohort [9]. Furthermore, research utilizing the dietary inflammatory index has demonstrated that diet-induced inflammation is associated with a higher likelihood of sarcopenia [10]. Some studies have indicated higher levels of pro-inflammatory cytokines, such as tumor necrosis factor (TNF)-α and interleukin (IL)-6, in individuals with sarcopenia [11, 12]. However, the results have not been consistent across different studies, and a meta-analysis conducted in 2017 found that the increase in TNF-α and IL-6 was not significant [13].

Sarcopenia patients are suggested to receive exercise interventions to restore muscle mass and function. Prior research has shown that aerobic exercise training can increase mitochondrial biogenesis in older individuals, resulting in improved muscle mass and strength [14]. Resistance exercise is also an important strategy for preventing muscle wasting, as it promotes muscle protein synthesis and mitigates degradation [15]. Currently, resistance training is recommended as the primary treatment for sarcopenia and has the additional benefit of reducing the risk of low-grade inflammation-related diseases such as atherosclerosis, obesity, and insulin resistance [16].

In addition to exercise, proper nutrient provision is crucial for treating sarcopenia. For example, a systematic review and meta-analysis have indicated that leucine supplementation, a branch-chain amino acid, can enhance muscle protein synthesis in older individuals and counteract age-related declines in muscle mass [17]. Another study involving individuals with both sarcopenia and vitamin D deficiency revealed that a vitamin D supplement of 10,000 IU, administered three times a week, led to improvements in muscle mass after six months [18]. Moreover, a cross-sectional analysis demonstrated a correlation between higher calcium intake and a lower risk of developing sarcopenia [19]. Thus, combining resistance exercise with appropriate nutritional support appears to be the most effective treatment approach for sarcopenia [15]. However, research on the inflammatory status following such interventions is limited. Therefore, we conducted a post-hoc analysis using data of sarcopenic patients from a randomized controlled trial (RCT) and non-sarcopenic participants from the same geriatric community cohort. Our study aimed to achieve two objectives: first, to examine whether specific pro-inflammatory cytokines are elevated in patients with sarcopenia; and second, to investigate any changes in these pro-inflammatory cytokine levels following exercise and nutritional support. In addition, body mass index (BMI), grip strength, and body composition were measured to diagnose sarcopenia and establish a baseline for comparison to ensure that the intervention was properly administered.

## MATERIALS AND METHODS

### Participant enrollment and criteria to diagnose sarcopenia

A group of older adults aged 65 years and above were selected as participants for a research project conducted at a geriatric clinic during their annual health check-up. In order to be included in the study, individuals had to meet certain criteria, including independent ambulation, comprehension of verbal instructions, and the ability to respond to the survey. The participants with cancer history and known unmanaged medical issues (like unstable angina) were not included.

**Figure 1:**
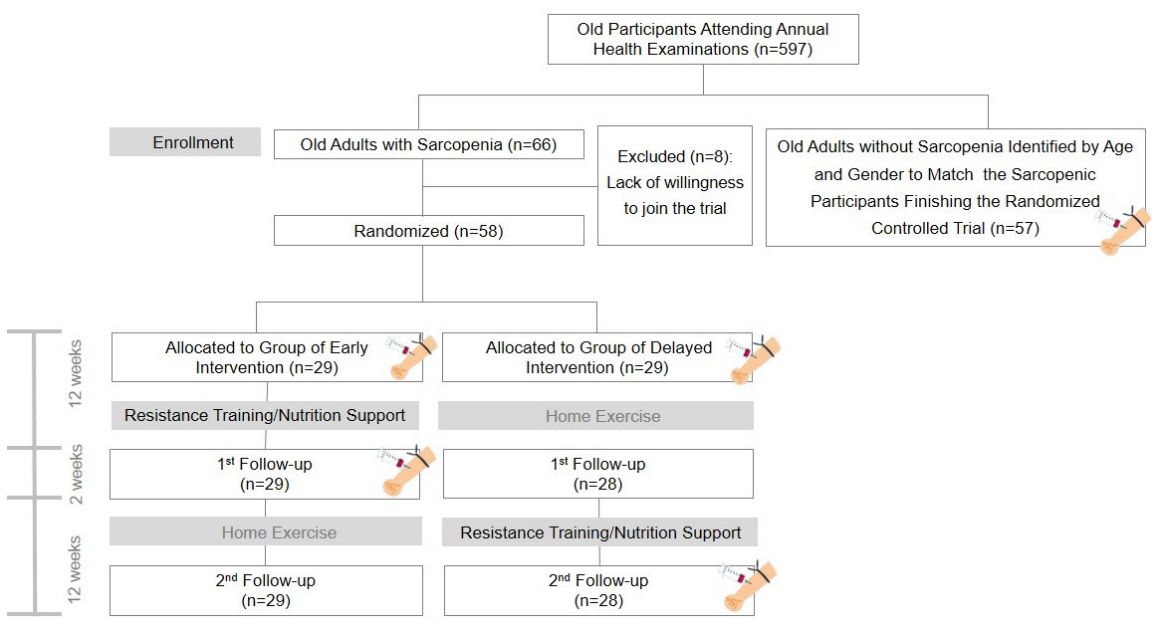
**Flow diagram of participant recruitment.** In this flow diagram, the blood drawing icon signifies the examination of pro-inflammatory cytokines.

Prior to participating in the study, all participants had been checked for their grip strength as well as body composition, and presented written informed consent. If the participant met the diagnostic criteria of sarcopenia, they were invited to join an RCT comparing the efficacy of early versus delayed exercise and nutritional interventions against sarcopenia. The RCT was registered on ClinicalTrials.gov under the identifier NCT02779088, with the registration completed on May 20, 2016. Further information about the trial is available at https://clinicaltrials.gov/ct2/keydates/NCT02779088. Non-sarcopenic control participants were matched for age and gender to sarcopenic participants from the same geriatric community cohort (Figure 1).

The diagnosis of sarcopenia followed the criteria established by the European Working Group on Sarcopenia in Older People (EWGSOP) consensus [20]. According to the EWGSOP guidelines, sarcopenic patients were defined through the presence of both low muscle strength and low muscle mass. The definition of low muscle mass was a skeletal muscle mass index below 7.40 kg/m^2^ for men and below 5.14 kg/m^2^ for women. Low muscle strength was defined as grip strength below 30 kg for men and below 20 kg for women. On the other hand, the control group comprised of older adults without low muscle mass and low muscle strength.

### Exercise intervention for sarcopenic patients

As a post-hoc analysis of an RCT [21] aimed to examine the efficacy of early versus delayed exercise and nutritional interventions against sarcopenia, serum inflammatory markers were assessed at the baseline and the end of a 12-week intervention period in the sarcopenic group. Both sets of participants in the RCT underwent a two-phase intervention program, each spanning a duration of 12 weeks with a two-week break in between. For the early intervention group, the initial phase encompassed the comprehensive intervention, followed by the home-based program. In contrast, for the delayed intervention group, the sequence was reversed, commencing with the home-based exercise phase, followed by the comprehensive intervention program. On the other hand, the control group did not receive any intervention and served as a baseline comparator for the sarcopenic group (Figure 1).

Participants diagnosed with sarcopenia were instructed to perform a 10-minute warm-up exercise, followed by three sets of 10 repetitions of leg press, leg extension, and leg curl, as part of the exercise intervention. These training sessions occurred twice per week. A 10-minute cool-down exercise involving bicycling was performed at the conclusion of each session.

Additionally, participants received nutritional supplementation during the intervention period. The supplementation regimen included daily consumption of two sticks of branched-chain amino acids (BCAA-Amino Vital Pro®, Ajinomoto, Tokyo, Japan) and two tablets of Caltrate supplement containing 600 mg of calcium and 800 IU of vitamin D3 (Pfizer, New York, NY, USA). A stick (3.6 g) of BCAA-Amino Vital Pro® encompassed 0.54 g of leucine, 0.43 g of isoleucine, 0.36 g of valine, 0.65 g of glutamine, 0.61 g of arginine, and 1.01 g of other amino acids.

### Outcome measures

### Determination of serum indicator level

In the morning, each participant underwent a fasting period of at least a few hours before a venous blood sample of 5 ml was collected from their elbow. Immediately after the blood draw, the blood biochemistry indicators were tested. The collected blood was then centrifuged at room temperature, at a speed of 4000 rpm for 8 minutes. For further analysis of inflammatory markers, an additional 400 μl of serum was extracted and divided into two Eppendorf tubes. These tubes were stored in a refrigerator at a temperature of -70° C for later testing. The levels of various markers, including TNF- α, IL-1β, IL-6 and IL-15 were quantified using competitive inhibition enzyme-linked immunosorbent assay (ELISA) kits (Thermo Fisher Scientific, Invitrogen, Waltham, MA, USA) [22]. The serum interleukin content was determined by measuring the optical density value at a wavelength of 450 nm using a spectrophotometer. Serum inflammatory cytokine measurements are a valid and reliable way to assess systemic inflammation. This has been demonstrated by studies comparing serum cytokine levels to cytokine levels in other tissues, such as bronchoalveolar lavage fluid (BALF), as well as by studies assessing the reliability of serum cytokine measurements over time. For example, a 2019 study found that serum levels of IL-6, IL-8, and TNF-α were strongly correlated with BALF levels of these cytokines in older adults, suggesting that serum measurements are a valid way to assess systemic inflammation [23]. Additionally, a 2011 study found that multiplex-based assays for measuring cytokine levels in serum and plasma were highly correlated with ELISA results for most of the cytokines tested, suggesting that they are a valid way to measure cytokine levels in serum and plasma [24]. Finally, a 2012 study found that serum levels of IL-6, IL-8, and TNF-α were highly reliable in healthy adults, with intraclass correlation coefficients (ICCs) greater than 0.70 [25].

### Measurement of grip strength and body composition

Grip strength assessment was conducted utilizing the hand dynamometer (Fabrication Enterprises Inc., Irvington, NY, USA) on the dominant hand. Participants were instructed to exert a strong and sustained grip on the device three times, with intervals of at least one minute between each trial. The highest recorded value from the three trials was considered as the participant’s grip strength [21]. The systematic review by Bohannon et al. [26] found that the Jamar dynamometer is a valid and reliable tool for measuring handgrip strength in adults of all ages. The validity of the Jamar dynamometer was assessed by comparing its results to other measures of handgrip strength, such as the results from a maximal isometric voluntary contraction (MIVC). The researchers found that the Jamar dynamometer produced results that were highly correlated with the results of the MIVC (ICC = 0.99). The reliability of the Jamar dynamometer was assessed by measuring handgrip strength multiple times on the same day and on different days. The researchers found that the Jamar dynamometer produced consistent results over time (ICC = 0.98).

To obtain body composition measurements, participants were instructed to observe an overnight fasting period of at least eight hours prior to the examinations. They were required to disrobe and wear gowns during the measurement process to ensure accuracy and consistency. A standard digital weight and height meter, with a precision of up to 100 grams in weight and 1 millimeter in height, was used to measure body weight and height. Body mass index (BMI) was calculated by dividing the weight in kilograms by the square of the height in meters (kg/m^2^). Whole-body dual-energy X-ray absorptiometry (DXA, Stratos dR, DMS Group, Paris, France) was employed to obtain comprehensive body composition data for the participants [21]. A comparative investigation [27] comparing DXA with multislice computed tomography showed a substantial alignment between the two techniques. The Bland-Altman analysis revealed strong agreement, with limits of agreement ranging from -1.56 to 2.54 kg for abdominal total tissue mass and -0.40 to 1.94 kg for abdominal fat mass. Furthermore, DXA displayed remarkable consistency when employed by three distinct operators to evaluate total, fat, and lean body mass in the L1-L4 region, as evidenced by ICCs of 0.94, 0.97, and 0.89, respectively.

### Time frame of measurements

Regarding BMI, grip strength, and body composition, we conducted measurements at three time points: baseline, the first follow-up, and the second follow-up for all participants in the RCT arm. However, for serum inflammatory cytokines, measurements were taken at baseline and either at the first follow-up for the early intervention RCT group or at the second follow-up for the delayed intervention RCT group. In contrast, the control arm, which did not undergo any intervention, had measurements obtained solely at the baseline assessment.

### Statistical analysis

For the RCT arm, only sarcopenic cases who completed both follow-up assessments were included in the analysis, consistent with the per-protocol approach. The Shapiro-Wilk test was used to assess the normal distribution of continuous variables, which were presented as mean ± standard deviation (SD) with a 95% confidence interval (CI). Depending on the normality of the distribution, between-group comparisons were conducted using either one-way analysis of variance (ANOVA) or the Mann-Whitney U test. Categorical data, presented as numerical values and percentages, were analyzed employing the chi-squared (for non-sparse data) or Fisher’s exact tests (for sparse data). Pearson’s correlation coefficient (r) was utilized to quantify the correlation between target biomarkers and grip strength as well as skeletal muscle mass index.

To evaluate the influence of exercise and nutritional intervention on target biomarkers in individuals with sarcopenia, a Kruskal-Wallis ANOVA was conducted. The association between selected target biomarkers (exhibiting significant differences between sarcopenic cases and controls) and variables such as age, gender, sarcopenia, and intervention were examined using the generalized estimating equation (GEE) [28]. To assess the ability of each cytokine to distinguish between sarcopenia and non-sarcopenia, we used receiver operating characteristic (ROC) curves. The optimal cutoff points were determined using the Youden index. The statistical analysis was performed using SAS version 9.4 (SAS Institute Inc., Cary, NC, USA) and MedCalc 14.0 (MedCalc Software, Ostend, Belgium), and statistical significance was determined with a two-tailed *p*-value threshold of less than 0.05.

### Data sharing statement

The data that support the findings are available from the corresponding author upon reasonable request.

## RESULTS

### Participant recruitment

The sarcopenic participants were identified from a geriatric community cohort attending annual health examinations (n = 597). Sixty-six sarcopenic patients were initially invited to join a RCT comparing early vs. delayed combined nutrition and exercise intervention for sarcopenia. Fifty-eight patients were willing to join the RCT with cytokine and other relevant parameters measured at baseline. One patient in the delayed intervention group was lost at the 1st follow-up, resulting in a dropout rate of 1.7% (1/58). A total of 57 sarcopenic patients with available cytokine data before and after intervention were analyzed along with 57 gender- and sex-matched participants without sarcopenia identified from the same geriatric community cohort (Figure 1).

### Baseline comparison between sarcopenic patients and controls

**Figure 2:**
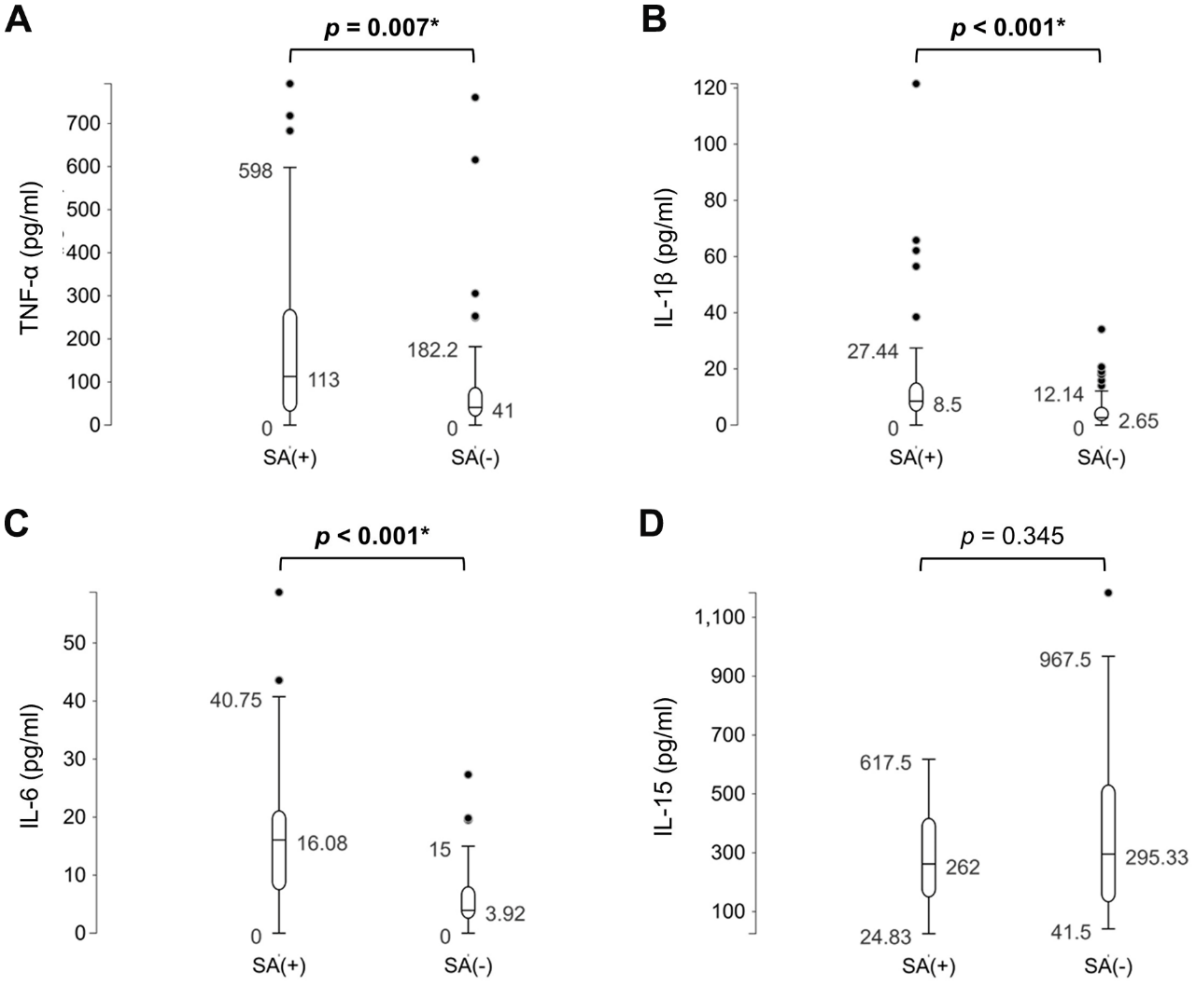
Comparisons of the levels of (**A**) tumor necrotizing factor (TNF)-α, (**B**) interleukin (IL)-1β, (**C**) IL-6, and (**D**) IL-15 between participants with and those without sarcopenia (SA). *, *p*<0.05.

**Figure 3:**
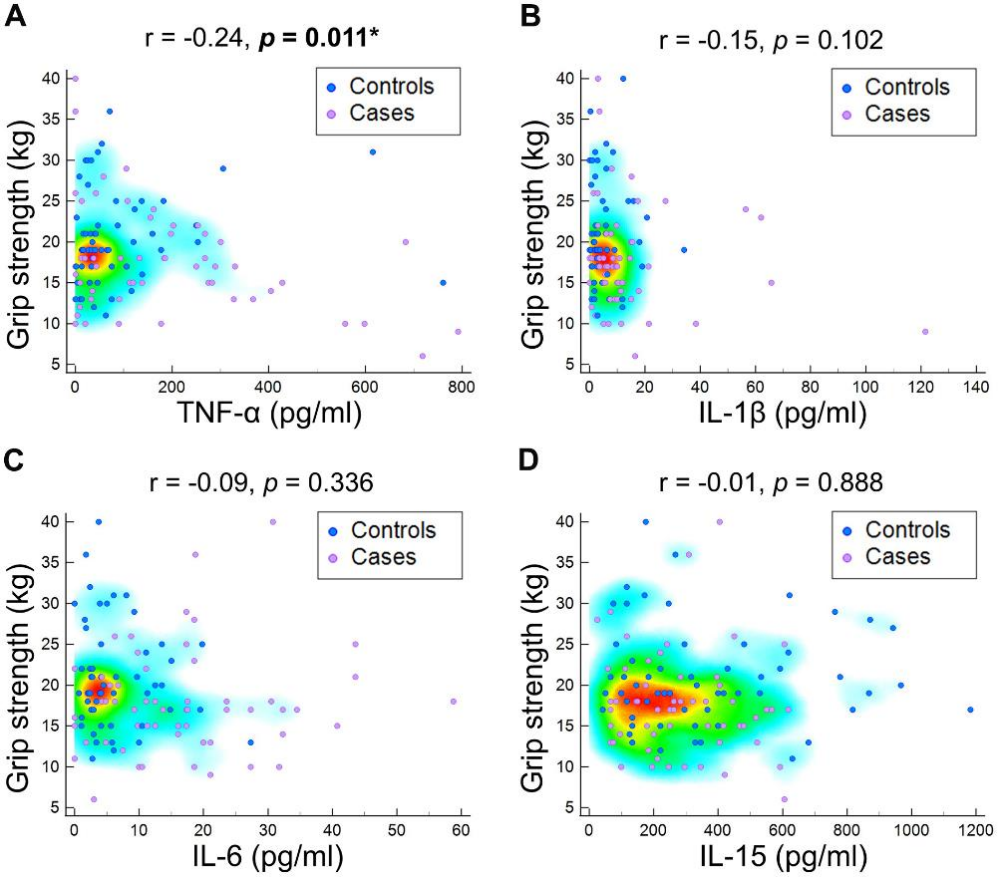
Correlation of grip strength at baseline with (**A**) tumor necrotizing factor (TNF)-α, (**B**) interleukin (IL)-1β, (**C**) IL-6, and (**D**) IL-15.

**Figure 4:**
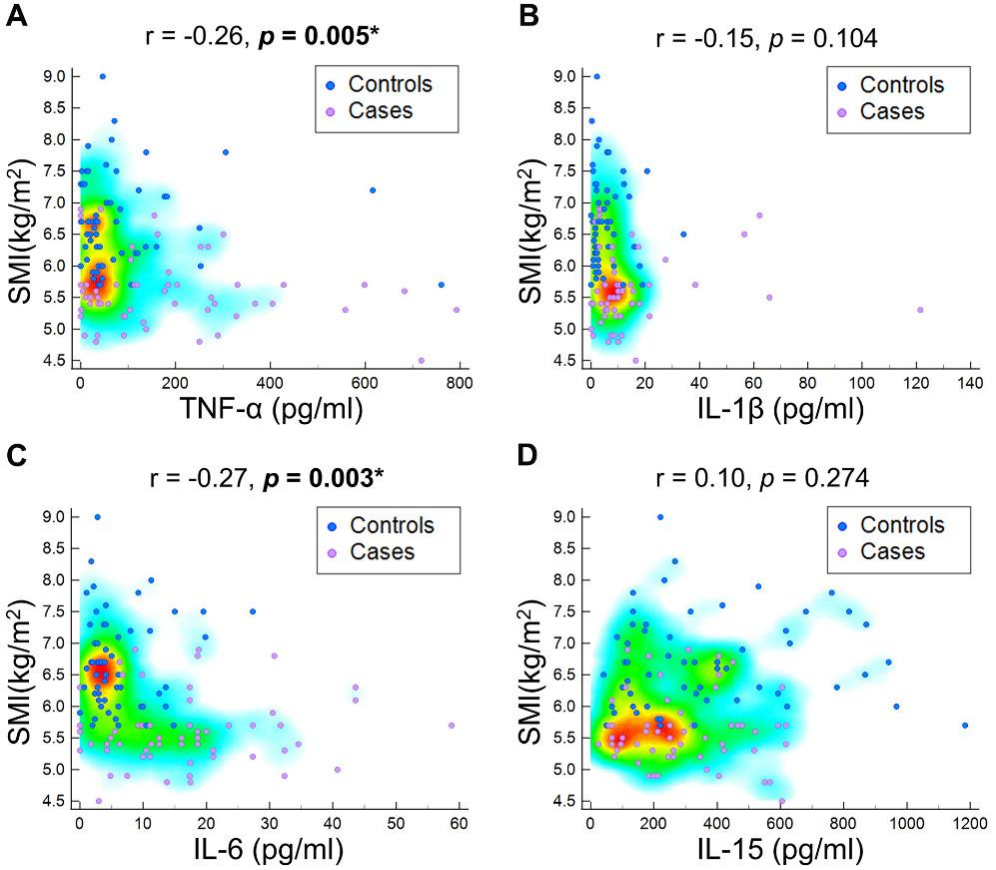
Correlation of skeletal muscle mass (SMI) at baseline with (**A**) tumor necrotizing factor (TNF)-α, (**B**) interleukin (IL)-1β, (**C**) IL-6, and (**D**) IL-15.

**Figure 5:**
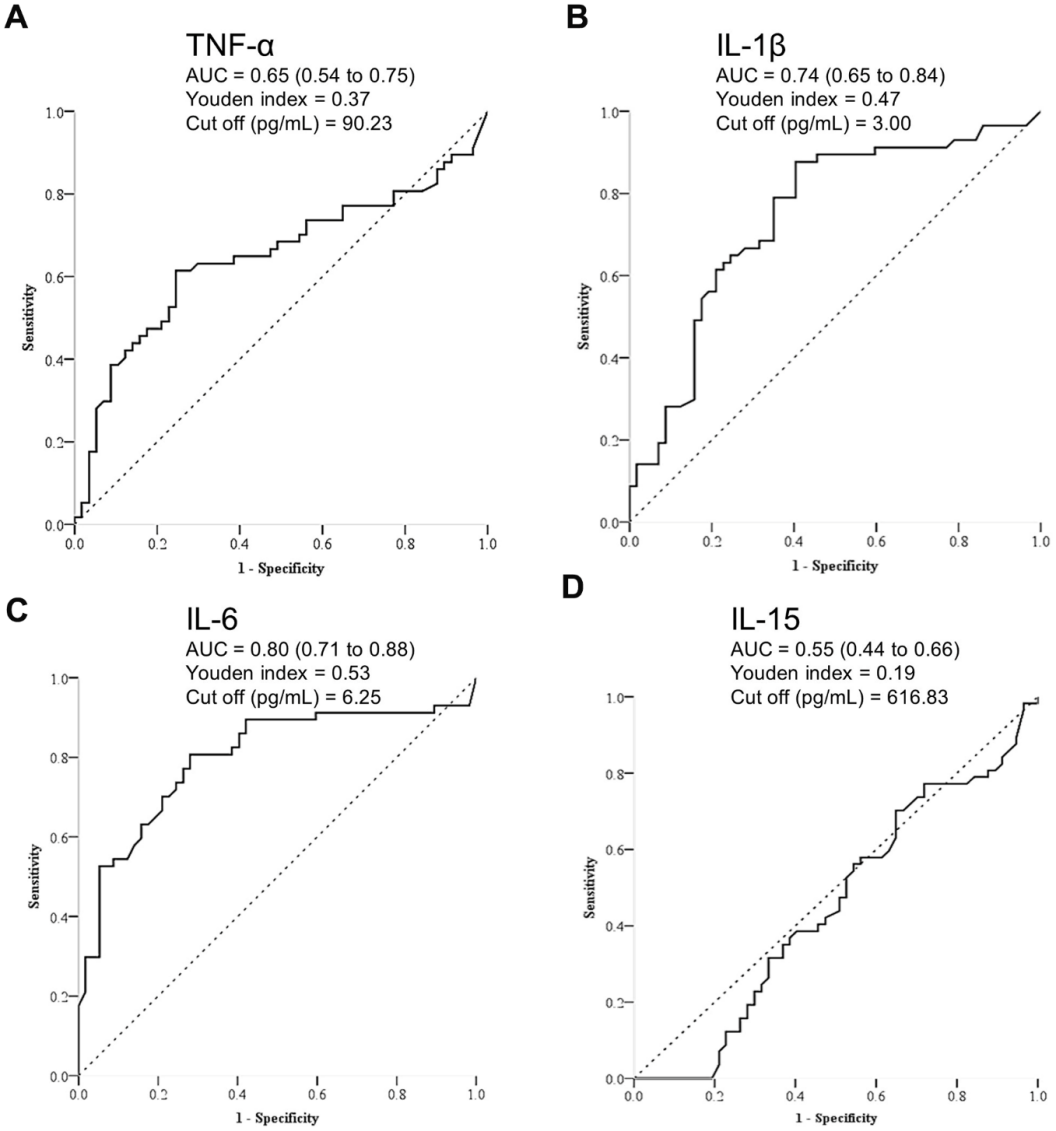
Receiver operating characteristic analysis for discriminating participants with and those without sarcopenia by (**A**) tumor necrotizing factor (TNF)-α, (**B**) interleukin (IL)-1β, (**C**) IL-6, and (**D**) IL-15.

The sarcopenic group exhibited significantly lower body weight (*p*<0.001), body mass index (*p*<0.001), grip strength (*p*=0.006), and skeletal muscle mass index (*p*<0.001) compared to the control group (Table 1). Furthermore, the sarcopenic patients had higher levels of TNF-α (*p*=0007), IL-1β (*p*<0.001) and IL-6 (*p*<0.001). There was no significant difference in IL-15 between participants with and those without sarcopenia (*p*=0.345) (Figure 2). Furthermore, in relation to the baseline values of pro-inflammatory markers in both the sarcopenic cases and controls, there was a negative correlation between TNF-α and skeletal muscle mass index (r = -0.26, *p* = 0.005), as well as grip strength (r = -0.24, *p* = 0.011). Additionally, IL-6 exhibited a negative association with skeletal muscle mass index (r = -0.27, *p* = 0.003) (Figures 3, 4). The area under curve of the ROC analysis to discriminate participants with and those without sarcopenia was 0.65 (95% CI, 0.54-0.75) for TNF-α, 0.74 (95% CI, 0.65-0.84) for IL-1β, 0.80 (95% CI, 0.71-0.88) for IL-6 and 0.55 (95% CI, 0.44-0.66) for IL-15 (Figure 5).

**Table 1:** Comparison of demographics, grip strength, skeletal muscle mass index and pro-inflammatory cytokines between participants with and those without sarcopenia.

	**Controls (n = 57)**	**Sarcopenic cases (n = 57)**	***p*-value**
**Demographic**			
Gender (female)	44 (77.2%)	44 (77.2%)	1.000
Age (year)	75.00 ± 5.87 (73.44-76.56)	75.02 ± 5.91 (73.45-76.59)	0.987
Height (cm)	155.12 ± 7.63 (153.10-157.15)	154.46 ± 7.04 (152.59-156.32)	0.629
Weight (kg)	61.82 ± 9.14 (59.40-64.25)	52.44 ± 7.42 (50.47-54.40)	<0.001*
Body mass index (kg/m^2^)	25.71 ± 3.61 (24.75-26.67)	21.92 ± 2.11 (21.36-22.48)	<0.001*
**Physical performance**			
Hand grip strength (kg)	20.95 ± 6.21 (19.30-22.59)	17.63 ± 6.36 (15.94-19.32)	0.006*
**Body Composition**			
Skeletal muscle index (kg/m^2^)	6.71 ± 0.74 (6.51-6.91)	5.61 ± 0.56 (5.46-5.76)	<0.001*
**Pro-Inflammatory cytokines**			
TNF-α (pg/mL)	86.70 ± 134.08 (51.12-122.28)	179.60 ± 194.19 (128.07-231.12)	0.007*
IL-1β (pg/mL)	5.26 ± 6.38 (3.57-6.95)	14.11 ± 19.88 (8.83-19.38)	<0.001*
IL-6 (pg/mL)	6.03 ± 5.48 (4.58-7.49)	16.80 ± 12.45 (13.50-20.11)	<0.001*
IL-15 (pg/mL)	365.77 ± 275.24 (292.74-438.80)	291.49 ± 174.25 (245.25-337.72)	0.345

* indicates *p* <0.05. The continuous variables are expressed by the mean and standard deviation (95% confidence interval of mean), whereas the categorical variables are shown by the number (percentage). TNF, tumor necrotizing factor; IL, interleukin.

### Effects of exercise and nutritional intervention

**Figure 6:**
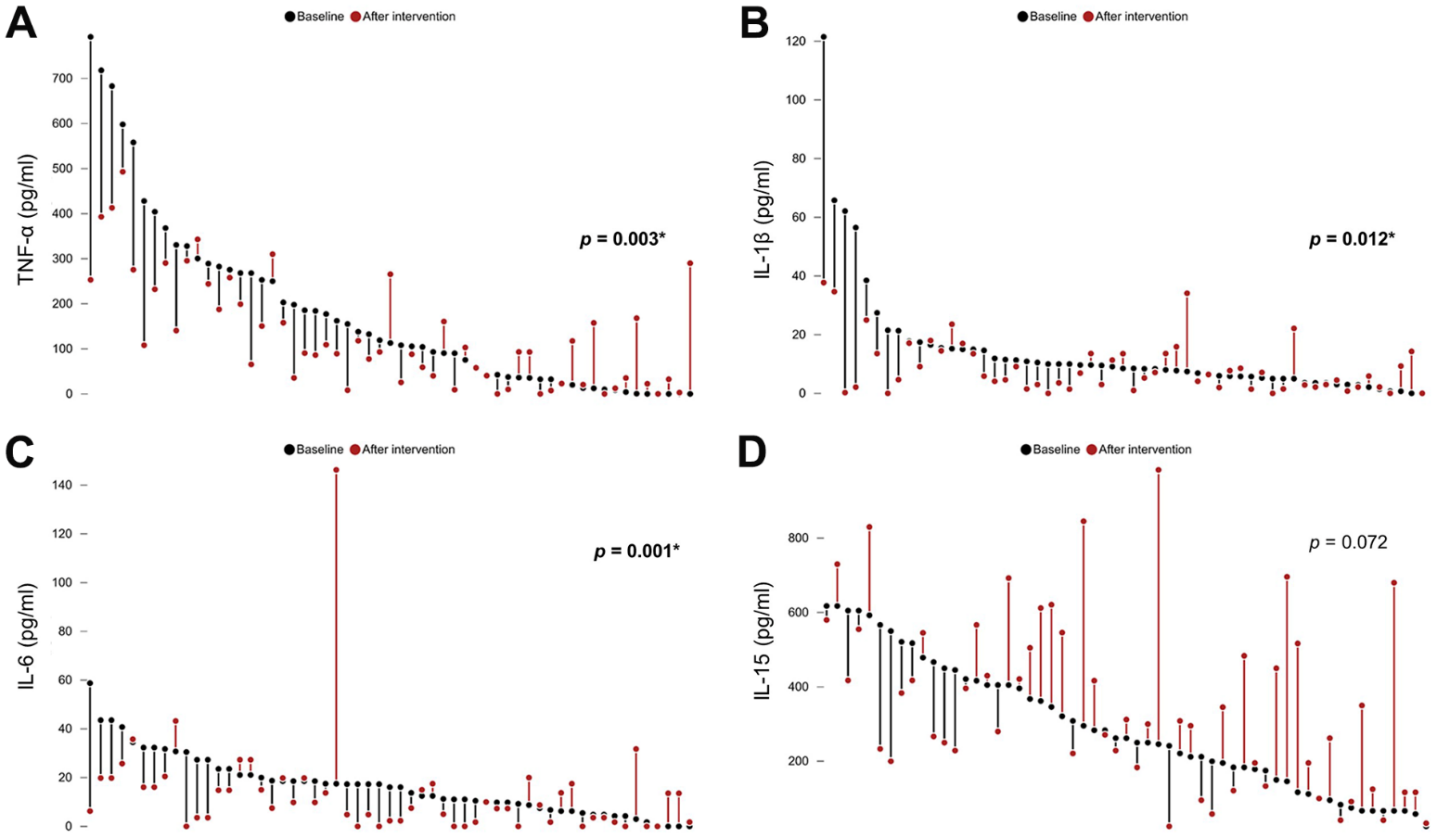
Comparisons of the levels of (**A**) tumor necrotizing factor (TNF)-α, (**B**) interleukin (IL)-1β, (**C**) IL-6, and (**D**) IL-15 among participants with sarcopenia before and after exercise and nutrition intervention. *, *p*<0.05.

Following the intervention, the sarcopenic group exhibited a significant improvement in grip strength and skeletal muscle mass index. Additionally, there was a noteworthy reduction in TNF-α (*p*=0.003), IL-1β (*p*=0.012) and IL-6 (*p*=0.001) levels compared to baseline measurements, whereas IL-15 was likely to elevate (*p*=0.072) (Table 2 and Figure 6). The GEE analysis adjusted by age and gender revealed sarcopenia significantly elevated TNF-α (*p*=0.003), IL-1β (*p*=0.001) and IL-6 (*p*<0.001), whereas intervention led to a decrease in TNF-α (*p*=0.005) and IL-1β (*p*=0.017) and an increase in IL-15 (*p*=0.024) (Table 3). No adverse events were reported after the exercise and nutritional intervention.

**Table 2:** Comparison of grip strength, skeletal muscle mass index and pro-inflammatory cytokines before and after exercise and nutrition intervention in patients with sarcopenia.

	**Before**	**After**	**Difference (After-Before)**	***p*-value**
**Physical performance**				
Hand grip strength (kg)	17.63 ± 6.36 (15.94-19.32)	20.53 ± 5.78 (18.99-22.06)	2.89 ± 4.80 (1.62-4.17)	<0.001*
**Body Composition**				
Skeletal muscle index (kg/m^2^)	5.61 ± 0.56 (5.46-5.76)	5.98 ± 0.75 (5.78-6.18)	0.37 ± 0.43 (0.26-0.48)	<0.001*
**Pro-Inflammatory cytokines**				
TNF-α (pg/mL)	179.60 ± 194.19 (128.07-231.12)	130.76 ± 121.04 (98.64-162.88)	-48.84 ± 130.8 (-83.55--14.12)	0.003*
IL-1β (pg/mL)	14.11 ± 19.88 (8.83-19.38)	8.72 ± 9.05 (6.32-11.13)	-5.38 ± 17.21 (-9.95--0.82)	0.012*
IL-6 (pg/mL)	16.80 ± 12.45 (13.50-20.11)	13.22 ± 20.56 (7.76-18.68)	-3.58 ± 21.87 (-9.39-2.22)	0.001*
IL-15 (pg/mL)	291.49 ± 174.25 (245.25-337.72)	356.93 ± 230.31 (295.82-418.04)	65.44 ± 221.2 (6.75-124.14)	0.072

* indicates *p* <0.05. The continuous variables are expressed by the mean and standard deviation (95% confidence interval of mean). TNF, tumor necrotizing factor; IL, interleukin.

**Table 3:** Association of the level of pro-inflammatory cytokine with ages in years, sex, sarcopenia and intervention using exercise and nutrition support analyzed using generalized estimating equation.

	**Age (Year)**	**Sarcopenia**	**Intervention**	**Female gender**
TNF-α (pg/mL)	1.80	92.86	-48.84	22.29
	(-3.38 to 6.98)	(32.34 to 153.39)	(-82.50 to -15.17)	(-49.91 to 94.49)
	*p =* 0.496	*p =* 0.003*	*p =* 0.005*	*p =* 0.545
IL-1β (pg/mL)	0.24	8.85	-5.38	0.11
	(-0.05 to 0.54)	(3.50 to 14.19)	(-9.81 to -0.96)	(-4.34 to 4.57)
	*p =* 0.107	*p =* 0.001*	*p =* 0.017*	*p =* 0.960
IL-6 (pg/mL)	-0.09	10.77	-3.58	-1.45
	(-0.42 to 0.24)	(7.28 to 14.27)	(-9.21 to 2.05)	(-6.01 to 3.11)
	*p =* 0.602	*p* < 0.001*	*p* = 0.212	*p =* 0.533
IL-15 (pg/mL)	-1.85	-74.25	65.44	-13.14
	(-9.12 to 5.41)	(-157.88 to 9.38)	(8.52 to 122.37)	(-112.97 to 86.69)
	*p =* 0.617	*p =* 0.082	*p =* 0.024*	*p =* 0.796

* indicates *p* <0.05. The values are shown by their β coefficients and 95% confidence interval. TNF, tumor necrotizing factor; IL, interleukin.

## DISCUSSION

The study yielded to several important findings. Firstly, it has been observed that patients with sarcopenia exhibit elevated levels of inflammatory cytokines such as TNF-α, IL-1β, and IL-6. However, interventions involving nutrition support and exercise have shown promising results in significantly reducing the aforementioned elevated cytokines.

In the present study, four kinds of inflammatory cytokines were examined, encompassing TNF-α, IL-1β, IL-6 and IL-15. TNF-α, primarily produced by macrophages and monocytes, initiates cellular apoptosis, regulates non-specific immune responses to counteract the spread and growth of infectious pathogens. Moreover, TNF-α acts as a strong inducer of nuclear factor kappa B (NF-κB), which plays a pivotal role in activating multiple pro-inflammatory genes within the cell nucleus. However, excessive and prolonged production of TNF-α can have detrimental effects, such as inducing joint destruction in patients with rheumatoid arthritis [29]. IL-1β, an intracellular cytokine belonging to the IL-1 family, plays a crucial role in coordinating innate and adaptive immune responses. It is primarily expressed in myeloid cells like monocytes, macrophages, and neutrophils. IL-1β is involved in protecting against bacterial and fungal infections, particularly in mucosal tissues, by regulating IL-17 production [30]. Additionally, it has been implicated in the development of conditions such as rheumatoid arthritis, ankylosing spondylitis, and juvenile idiopathic arthritis [30]. IL-6 is a versatile cytokine with crucial roles in host defense. Its level increases significantly during inflammation but remain low under healthy conditions. IL-6 is implicated in the transition from acute to chronic inflammation, leading to tissue damage. Elevated levels of IL-6 and its receptors are commonly observed in patients with rheumatoid arthritis [31]. IL-15 is produced mainly by monocytes, macrophages, and skeletal muscle cells. It also exhibits immune-regulatory effects on various immune cells, including nature killer cells, T lymphocytes, neutrophils, and monocytes. Finally, IL-15 functions as a myokine and affects other tissues such as the brain, bone, skeletal muscle, and adipose tissue. It promotes skeletal muscle anabolism and protects against visceral adiposity [32].

In our study, three (TNF-α, IL-1β, and IL-6) of four cytokines we examined revealed elevated in sarcopenic patients compared with the controls. An animal study using the sarcopenic mice model found that TNF-α contributed to sarcopenia by mediating pyroptosis, one type of programmed cell death, through activating the pathways of caspase-8 and caspase-3 [33]. Dupont et al. conducted a cross-sectional analysis enrolling 40 sarcopenic patients, showing TNF-α to be inversely correlated with the 36-Item Short Form Survey (SF-36) physical component score in men [34]. Furthermore, Parker et al. observed a noteworthy increase in IL-1β expression in fibro-adipogenic progenitors following a 2-week immobilization of hindfeet in an animal model, as evidenced by RNA sequence data [35]. These fibro-adipogenic progenitors are a specialized type of stem cells found within muscle tissues and have been discerned for the important role of muscle regeneration and hypertrophy. Lastly, in a cohort consisting of 164 participants aged between 61 and 90 years, it was observed that individuals with sarcopenia exhibited a higher risk of malnutrition and higher levels of IL-6 compared to those without sarcopenia in the initial univariate analysis [11]. After accounting for potential confounding factors through adjustment, the presence of sarcopenia was found to have a positive correlation with IL-6. Our study result was in line with the previous animal and clinical studies, highlighting sarcopenia to be a pro-inflammatory status leading to elevated TNF-α, IL-1β, and IL-6.

However, our research findings revealed no significant increase in IL-15 levels among patients with sarcopenia compared to the non-sarcopenic controls. Interestingly, we observed that exercise and nutritional interventions resulted in elevated IL-15 levels in the sarcopenic patients. A study involving 160 older adults provided insights into our results, demonstrating that control subjects had significantly higher levels of IL-15 compared to individuals with sarcopenia [36]. Moreover, in the multivariate regression analysis, plasma IL-15 levels were found to be independently and inversely associated with sarcopenia. This suggests that a lower level of IL-15 could impair the anabolic effects on skeletal muscles and hinder myoblast differentiation, potentially leading to the development of sarcopenia.

A notable discovery has been made regarding the reduction of TNF-α, IL-1β, and IL-6 levels following exercise and nutritional support in individuals diagnosed with sarcopenia. While the positive effects of exercise on diminishing pro-inflammatory cytokines have been observed in various populations, there has been limited research exploring the relationship between exercise and pro-inflammatory cytokines specifically in patients with sarcopenia. Koh et al. conducted a RCT to examine the effect of 4-week moderate intensity walking exercise on 27 obese patients [37]. They found that TNF-α significantly decreased in the exercise group but remained unchanged in the control group. Balducci et al. conducted a study to examine the impact of various types of exercises on inflammatory serum biomarkers in patients diagnosed with type 2 diabetes and metabolic syndrome [38]. The researchers discovered that individuals who engaged in high-intensity aerobic and resistive exercises experienced reduced levels of TNF-α and IL-1β. Amin et al. investigated 18 Egyptian male handball players, comparing their serum IL-6 and TNF-α before and after intermediate-to-high intensity exercise [39]. Post-exercise downregulation of both cytokines was observed, coupled with an increase in the white count level.

We speculated moderate-to-high intensity exercise could reduce pro-inflammatory cytokine in the sarcopenic patients through several possible mechanisms. Regular exercise plays a significant role in reducing body fat, which is often elevated in individuals with sarcopenia. Adipocytes are a prominent source of pro-inflammatory cytokines. However, exercise-induced reduction in pro-inflammatory cytokines can occur independently of weight loss [38, 40]. Additionally, regular exercise has been shown to increase the concentration of circulating anti-inflammatory cytokines, such as IL-4, IL-10, and IL-13 [40]. Furthermore, resistance training is particularly effective in stimulating muscle protein synthesis and promoting muscle growth. Increased skeletal muscle mass has been found to have an anti-inflammatory effect as it releases myokines, which possess the ability to counteract the detrimental effects of pro-inflammatory cytokines [40].

In the present study, we hypothesized that nutrition supplements could potentially contribute to the reduction of circulating pro-inflammatory cytokines. A recent review has highlighted amino acids as endogenous metabolite modulators that may be beneficial in reducing lobular inflammation in non-alcoholic fatty liver disease [41]. In support of this notion, Tabesh et al. conducted a RCT involving 118 diabetic patients [41]. The study found that supplementation with 1000 mg/day of calcium alone, 50000 IU/week of vitamin D alone, and a combination of both resulted in a significant reduction of IL-6. Based on these findings, we believe that providing branched-chain amino acids, calcium, and vitamin D3 in conjunction with exercise training could have a synergistic effect in reducing the pro-inflammatory status of our sarcopenic patients.

In our current study, we observed a notable improvement in grip strength and muscle mass following a combined nutrition and exercise intervention. This finding aligns with the existing body of evidence supporting the efficacy of such interventions in treating sarcopenia. For instance, a comprehensive network meta-analysis conducted in 2021 [42], which examined 26 trials to compare the effects of exercise, nutrition, and their combination on muscle mass, muscle strength, and physical performance in older adults with sarcopenia, reported significant gains in handgrip strength (2.03 kg, 95% CI: 1.10-2.97) when exercise and nutrition were combined, as compared to a control group. Furthermore, a systematic review [43] carried out in 2023 emphasized the synergistic benefits of combining exercise and nutrition. It highlighted not only improvements in the primary outcome but also enhancements in other aspects, including strength, speed, stability, and various indicators of quality of life. Our own analysis confirmed that the increases in muscle mass and strength were associated with reductions in pro-inflammatory cytokines, providing further support for the multifaceted advantages of combined nutrition and exercise interventions in managing sarcopenia.

The present study has several limitations that should be acknowledged. Firstly, in the control group where no intervention was administered, we did not periodically assess their pro-inflammatory cytokine levels. Without a reference group for comparison, it is possible that the changes observed in cytokine levels among sarcopenic patients might have occurred as part of a natural recovery process. Secondly, the blood samples were collected within one week after the cessation of exercise training and nutritional support, preventing us from determining the duration of the post-intervention anti-inflammatory effect in our study. Thirdly, there are other imaging modalities for evaluating muscle mass in the diagnosis of sarcopenia, besides DXA. In recent years, ultrasound imaging has gained prominence as a muscle mass measurement tool [44] due to its excellent portability, lack of radiation, and incorporation in algorithms for diagnosing sarcopenia [45, 46]. The interplay between muscle mass measured by ultrasound imaging and changes in cytokine levels would be of clinical interest, and future studies are needed to investigate this relationship. Fourthly, there may be a potential association between sarcopenia and RAS dysregulation [47]. However, our study did not investigate whether RAS dysregulation changed in accordance with cytokine levels. The benefit of exercise-nutrition combination intervention could be partially attributed to the correction of RAS dysregulation, which also warrants more research to validate. Fifthly, in the present study, the stick for BCAA-amino acid supplementation did not exclusively include hydroxymethylbutyrate (HMB). As HMB has been shown to be beneficial for restoring muscle mass [48], it could be of clinical interest to exclusively use HMB as the main amino acid supplement in future investigations. Sixthly, the sarcopenic patients in our study received combinational interventions, including BCAA, vitamin D, calcium, and resistance exercise. Therefore, the anti-inflammatory effect cannot be explicitly attributed to any of these interventions.

## CONCLUSIONS

The study discovered that patients with sarcopenia exhibit elevated levels of TNF-α, IL-1β and IL-6. However, interventions involving nutrition support and exercise show promise in reducing these elevated cytokines. It is important to note that without intervention on the control group and periodic assessment of pro-inflammatory cytokine levels, the observed changes may be part of a natural recovery process. Additionally, the timing of blood sample collection limits our understanding of the post-intervention anti-inflammatory effects. Further research is necessary to fully evaluate the impact of these interventions on cytokine levels in sarcopenic patients.
